# The trilateral link between anaesthesia, perioperative visual loss and Flammer syndrome

**DOI:** 10.1186/s12871-016-0176-3

**Published:** 2016-02-04

**Authors:** Rossiana I. Bojinova, Katarzyna Konieczka, Peter Meyer, Margarita G. Todorova

**Affiliations:** 1 Department of Ophthalmology, University of Basel, Mittlere Strasse 91, CH-4031 Basel, Switzerland; 2 University of Montreal, Montreal, Canada

**Keywords:** Perioperative visual loss, Flammer syndrome, Autoregulation, Ocular blood flow, Anesthesia, Ocular/nonocular surgery, Doppler ultrasonography, Velocity, Resistance index, Plasma Endothelin-1 level

## Abstract

**Background:**

A variety of factors have been linked to perioperative visual loss during or directly after nonocular and ocular surgeries. Prolonged immobilization, biochemical factors and hemodynamic instability have been discussed as factors in the pathogenesis of this devastating complication. Perioperative visual loss in four consecutive patients, all featuring Flammer syndrome, is reported herein. To our knowledge, we present the first case series, which associates perioperative visual loss with Flammer syndrome. We assume that a low perfusion pressure, disturbed autoregulation of the ocular blood flow and altered drug sensitivity in such subjects, play significant role in the pathogenesis of this dreaded complication.

**Cases presentation:**

We analysed the medical records of four consecutive patients with permanent perioperative visual loss and complemented our findings with additional history taking and clinical examinations. A variety of tests was performed, including colour Doppler ultrasonography of the retroocular vessels, static and dynamic retinal vessel analysis. The visual loss was unilateral in three patients and bilateral in one. An extensive review of published perioperative vision loss cases was conducted.

All four patients were male Caucasians, and exhibited prominent signs and symptoms of Flammer syndrome. The visual loss originated from a propensity for unstable ocular blood flow, combined with hyperreactivity toward pharmacological stimuli, leading together to disturbed autoregulation of the blood supply, and subsequently - to ocular hypoxia. An identified intrinsic hypoperfusion diathesis was a crucial pathophysiologic link in all of the patients. Other, yet unknown systemic or local factors may also be involved in this process.

**Conclusions:**

A review of numerous publications of perioperative visual loss and our data, support our hypothesis for a novel pathophysiologic model and incorporate Flammer syndrome as a distinct risk factor for paradoxical visual loss, during nonocular and ocular surgeries, or invasive procedures. To prevent the complications produced by disturbed blood flow autoregulation in such patients, guidelines for screening and tailored preoperative approach are given.

## Background

Presumed pathogenic factors for perioperative visual loss, reported with a variety of nonocular surgeries, thus far include prolonged immobilization in prone position, external pressure on the eye, significant blood loss and hypotension, or excessive hydration with systemic hypertension and/or hemodilution, systemic hypothermia, as well as hyperlipidemia, most of which occurring usually in adults [1–3].

Our work highlights Flammer syndrome (FS) as a risk factor for visual loss during nonocular surgery in one adolescent patient and one adult patient, and during ocular interventions in two adult patients.

We conducted a retrospective study on four consecutive patients, presenting in our clinic, between December 2006 and December 2015, with persistent perioperative visual loss not explained by prevailing ophthalmic diseases.

The patients’ cases were indentified through thorough search in our multidisciplinary clinical meeting reports, produced in this nine-year period. In the files thus examined, we reviewed all data, along with the questionnaire used in cases of suspicion for disturbed vascular autoregulation. The questionnaire identifies signs and symptoms associated with FS. The inclusion criteria for our study were: positive answers to at least four out of ten signs or symptoms, and/or identified increased sensitivity toward medication. The exclusion criteria were: inclusion criteria not satisfied or a visual loss unequivocally due to purely ophthalmologic genesis.

In each case, a comprehensive evaluation, including extensive file review, history taking, full general and ophthalmologic examinations, and complete vascular and pharmacological workup were performed. Appropriate follow up was made and the findings were recorded each time.

Visual field examination, Dynamic Colour Doppler imaging of the orbital vessels and retinal vessel analysis were essential tools in the investigative process.

Plasma Endothelin-1 (ET-1, a peptide with potent systemic vasoconstricting effect) level was investigated at the time when a common profile of our group of patients began to point toward vascular dysregulation in the context of Flammer syndrome. The ET-1 measurement was obtained according to our standard protocol: the blood sample was drawn at room temperature through a venesection, following 30 minute rest in supine position, then the blood was immediately transferred to an EDTA containing tube and was centrifuged for 5 minutes at 4 degree Celsius (°C). The plasma was separated from the blood cells at 4 °C and kept at −80 °C until the essay was performed. The ET-1 plasma level was determined by radioimmunoassay with synthetic human/porcine ET-1 (Sigma Chemical Co), a rabbit antibody against synthetic ET (Peninsula Laboratories) and 125I-ET-1 (Amersham) [4].

## Case presentation

### Patient 1: an adolescent male, who suffered profound, permanent, unilateral vision loss in the perioperative period of a corrective scoliosis surgery

**Fig. 1:**
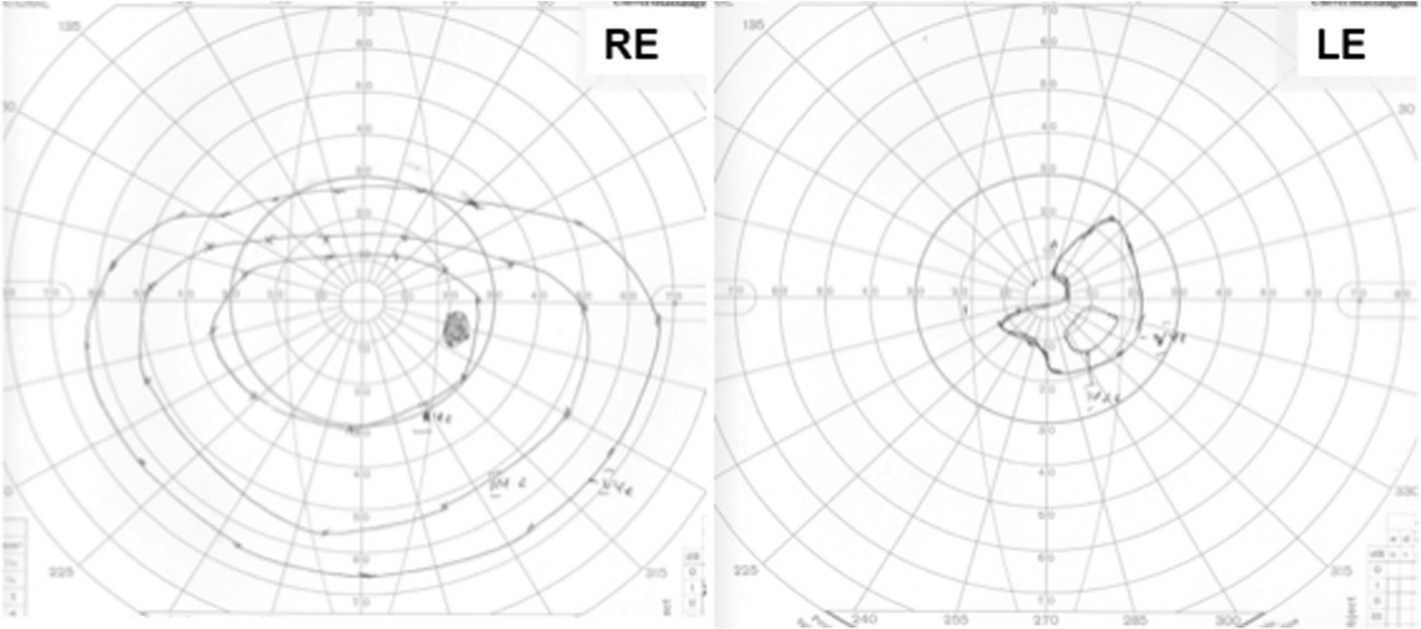
Goldmann perimetry Patient 1, Left eye (*LE*), shows a massive concentric visual field constriction with a remaining small isle of residual light perception in the paracentral inferior nasal visual field. Right eye (*RE*) is normal, except for an artefact due to droopy eyelid after the anaesthesia, which resulted in a temporary obstruction of the superior visual field

A 15 year-old patient with Body mass Index of 14.88 kg/m^2^ and unremarkable preoperative circulatory status, underwent 8 hour corrective scoliosis surgery, with 1400 ml blood loss. Upon awakening he noted blindness in his left eye. A profound visual loss, visual field constriction (Fig. 1) and reduced intraocular pressure (IOP) were documented. An examination showed relative afferent pupillary defect and ischemic retina in the left eye. A postoperative hypertensive state with blood pressure of 180/80 mmHg within a postoperative pain syndrome was documented.

An ultrasound study of his heart, 6 months prior, was unremarkable. The family history was significant for scoliosis in two-generations and uncomplicated scoliosis surgery of his twin-sister was performed the same day.

A postoperative retinal vessel analysis confirmed reduced venous vasodilation to flickering light in the left eye. His plasma Endothelin-1 level was 2.9 pg/ml (upper limit of the reference interval for our laboratory: 2.5 pg/ml). Nailfold capillary microscopy with a cold-provocation test yielded blood flow cessation in one out of four capillaries for 57 seconds (normal result: less than 11 seconds), while the patient was also positive for nine out of ten criteria for FS (Table 2) [5]. Twenty-four hour systemic blood pressure monitoring, yielded a mean blood pressure of 122/82 mmHg. The postoperative MRI and CT studies were unremarkable.

Doppler ultrasonography recorded a few months after the scoliosis surgery revealed reduced perfusion velocities in the left central retinal vein and artery, as well as in some cilioretinal arteries of both eyes, compared to the normal values (Table 1). Hence, in line with the systemic nature of the vascular dysregulation, the ciliary arteries of the uninvolved right eye also showed reduced velocities, while the measurements from the right central retinal vessels were consistent with the systemic compensatory efforts. We documented a decrease of the Resistance Index (RI) in the left central retinal artery and an increase of RI in the left cilioretinal arteries (Table 1). The RI calculations are based on the maximum Doppler shift waveforms. Just above normal were the minimal and maximal velocities in the right central retinal vein, while the systolic velocity in the right retinal artery was marginally elevated.

**Table 1:** Dynamic colour doppler imaging of the orbital vessels of patient 1

Color doppler imaging	Right eye		Normal values	Left eye	
Ophthalmic artery	16°			30°	
Systolic^1^	34.7		32.7–49.1	35.9	
Diastolic^2^	7.0		5.4–13.0	8.3	
RI^3^	0.80		0.70–0.85	0.77	
Central retinal artery	16°			5°	
Systolic	13.4		9.0–14.1	**4.3**	
Diastolic	2.9		2.1–4.7	**2.0**	
RI	0.78		0.63–0.78	**0.53**	
Central retinal vein					
MAX	−6.4		−6.1 to −3.1	**−1.9**	
MIN	−4.7		−4.6 to −2.3	**−1.7**	
Ciliary arteries	Lateral 27°	Medial 10°		Lateral 23°	Medial 19°
Systolic	9.3	**7.0**	9.2–14.4	**14.7**	**8.8**
Diastolic	**2.1**	**2.1**	2.2–5.3	2.7	**1.9**
RI	0.77	0.71	0.60–0.77	**0.81**	**0.79**

^1^Peak systolic velocity

^2^End diastolic velocity

^3^Resistance index

The values out of range are displayed in **bold**.

### Patient 2: an adult male with a visual loss, diagnosed right after uncomplicated pars plana vitrectomy on the same side, accompanied by temporary constriction of the visual field in the contralateral eye

**Fig. 2:**
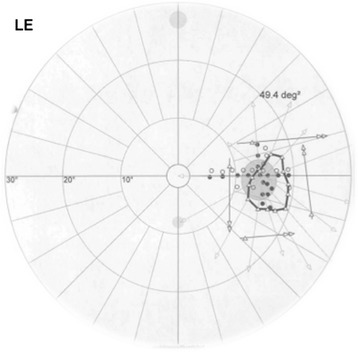
Octopus kinetic perimetry Patient 2, Left eye, shows a massive concentric visual field constriction with a remaining small isle of residual light perception in the peripapillary temporal visual field

**Fig. 3:**
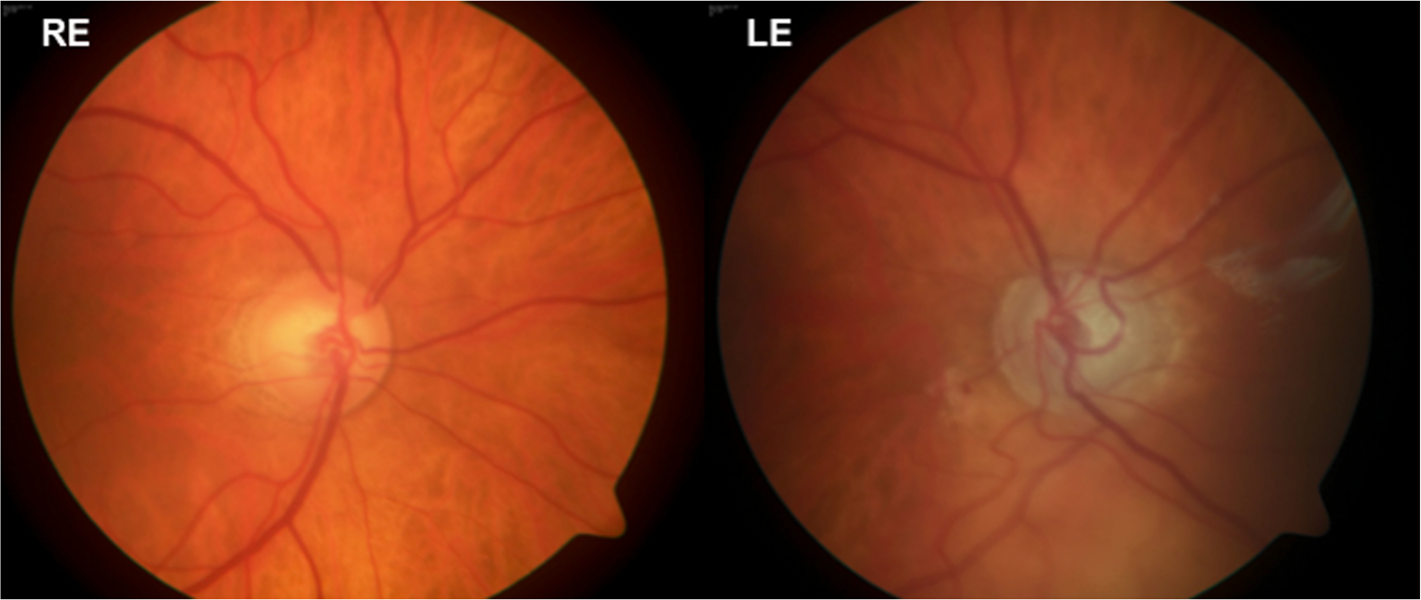
Patient 2, Posterior pole fundus imaging (30°) of both eyes showing pale optic disc of the left eye

Immediately after uncomplicated pars plana vitrectomy for retinal detachment in the left eye, a 71 year-old male patient noted a visual loss in his left eye (Fig. 2 and Fig. 3) and a temporary constriction of the temporal visual field in his right eye. The optic nerve head in the left eye was initially swollen, then turned pale. The anaesthesia consisted of retrobulbar injection of Mepivacaine before the surgery. The postoperative MRI showed no signs of cerebral ischemia or compression that could explain the visual loss.

However, a Retinal Vessel Analysis showed in both eyes reduced dilation of the arteries and veins, in response to flickering light. Dynamic Colour Doppler Imaging showed reduced systolic and diastolic perfusion velocity in the right central retinal artery, right central retinal vein, and in ciliary arteries. In addition, the patient had six out of ten positive criteria for Flammer syndrome (Table 2).

**Table 2:** Questionnaire items used to assess for signs and symptoms of Flammer syndrome

Signs and symptoms of Flammer syndrome		Patient 1	Patient 2	Patient 3	Patient 4
1	Do you suffer from cold hands or feet even in summer time?	+	+	+	+
2	Do you have a low blood pressure?	+	–	+	–
3	Are you less thirsty than others?	+	+	–	–
4	If you have to take medications, do you have the feeling that you react strongly to them?	+	Not known	Not known	+
5	Do you suffer from migraines?	+	Not reported	–	Not reported
6	Do you suffer from tinnitus (ringing in your ears)?	Not reported	+	+	+
7	Do you often feel cold when you are not moving for sometime?	+	+	+	+
8	Do you need a relatively long time to fall asleep, especially when you are cold?	+	+	+	+
9	Do you identify smells better than others?	+	Not reported	+	Not known
10	Have you noticed reversible skin blotches (white or red) when you were excited or angry?	+	+	+	+

+means sign/symptom present

–means sign/symptom absent

All data were consistent with a low perfusion pressure in the eye, in the presence of disturbed autoregulation, particularly of the Optic nerve head (ONH). Mepivacaine is a known vasoconstrictor of retrobulbar vessels [6]; prominent here, is the potential hypersensitivity to medication, of subjects with Flammer syndrome.

### Patient 3: an adult male with unexpected visual loss after peribulbar steroid application, in which physical injury to the eye was ruled out

**Fig. 4:**
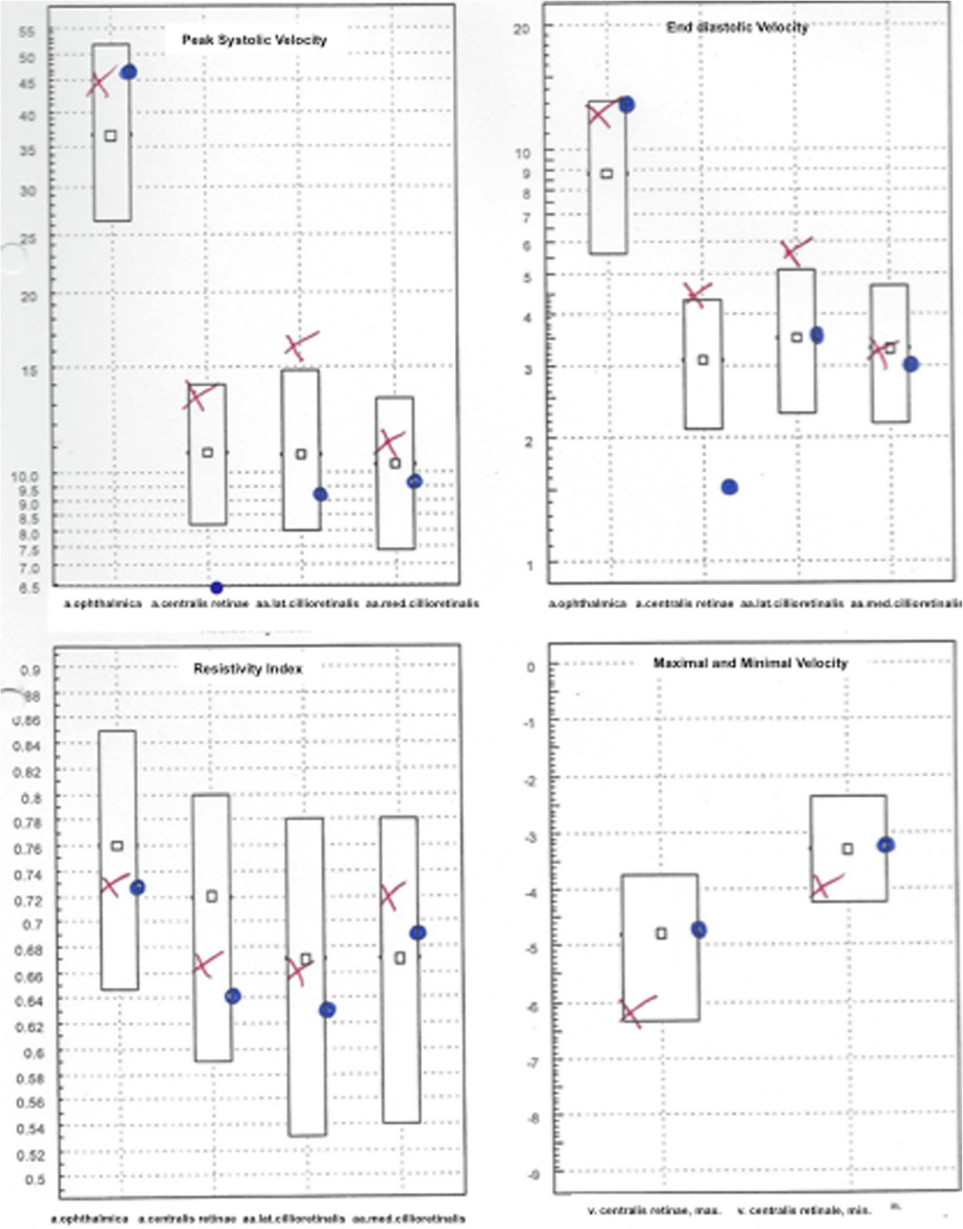
Patient 3, Dynamic Colour Doppler ultrasonography of both eyes showing reduced peak- systolic and end-diastolic velocities of the central retinal artery. The results are presented in box-plots. The right eye results are plotted as red crosses (x) and those of the left eye - as blue dots (°)

A 75-year-old gentleman suffered from reduced visual acuity (0.1) due to a persistent macular edema in the context of a chronic uveitis in his left eye. Peribulbar injection of betamethasone acetate - betamethasone sodium phosphate was performed to treat the condition. Unfortunately, immediately following the uncomplicated peribulbar injection, the patient reported “seeing only white”. His visual acuity was reduced to light perception. The ultrasound examination of the involved eye and the optic nerve showed neither signs of compartment syndrome, nor a direct injury of the eye or the optic nerve. Dynamic Colour Doppler-Ultrasound revealed reduced flow velocity in the left central retinal artery, whereas the flow in the ophthalmic- and cilioretinal arteries was symmetrically normal (Fig. 4). This patient suffered from FS, as well. In such patients not only cold, but also a mechanical stress can provoke a vasospasm. The peribulbar depot-steroid application may have compressed mechanically the ophthalmic artery, which is supplying the central retinal artery; although, it is also possible that the mechanical stress provoked a vasoconstriction.

### Patient 4: an adult male patient who exhibited bilateral visual loss, un-doubtfully connected to Catecholamine (Noradrenalin) use during cardiac surgery for Aortic Valve Replacement

**Fig. 5:**
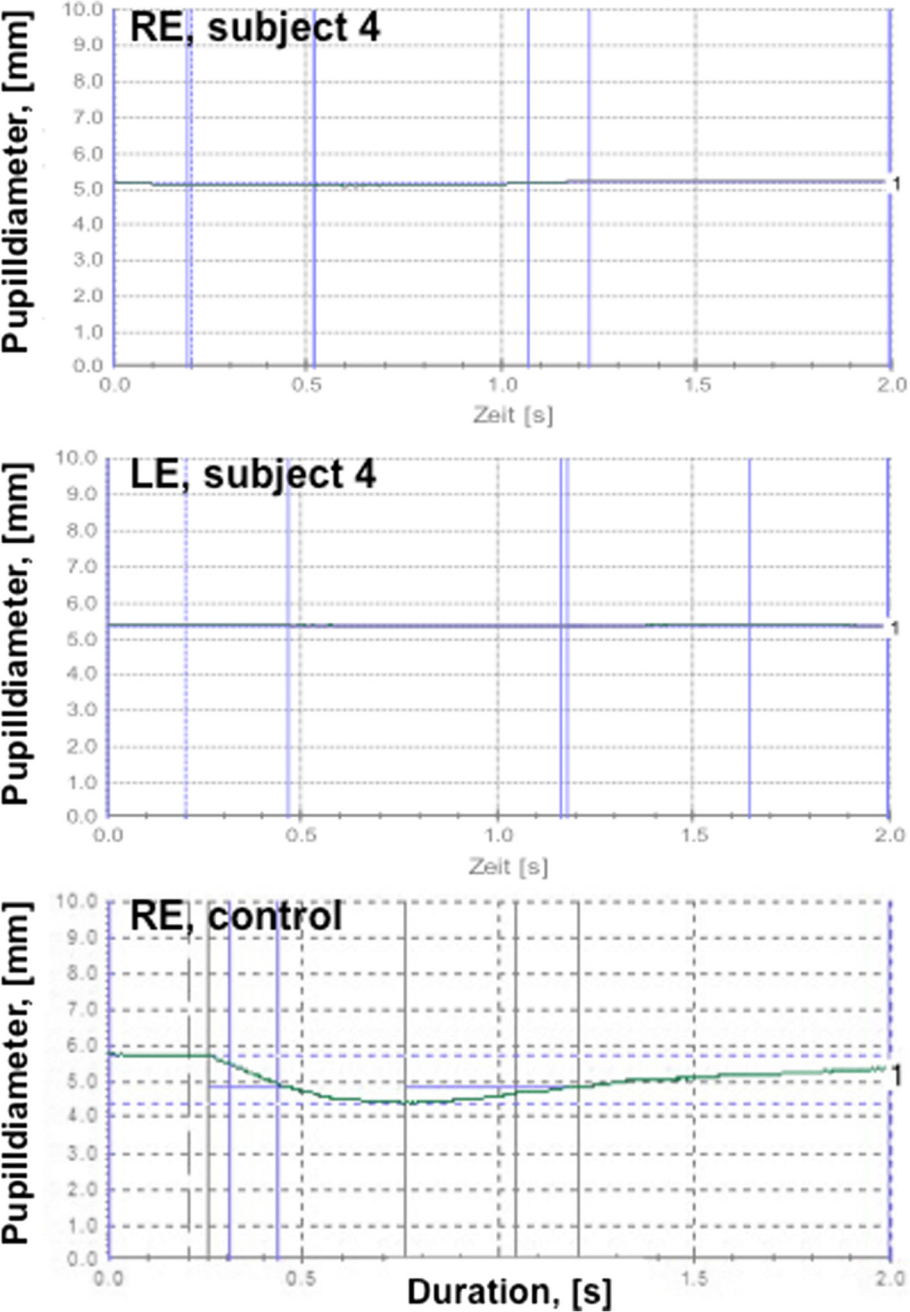
Patient 4, Pupillometry test results show afferent pupillary defect bilaterally (no change of the pupil diameter). For comparison, pupillometry test results of the right eye in a control subject are plotted at the bottom

**Fig. 6:**
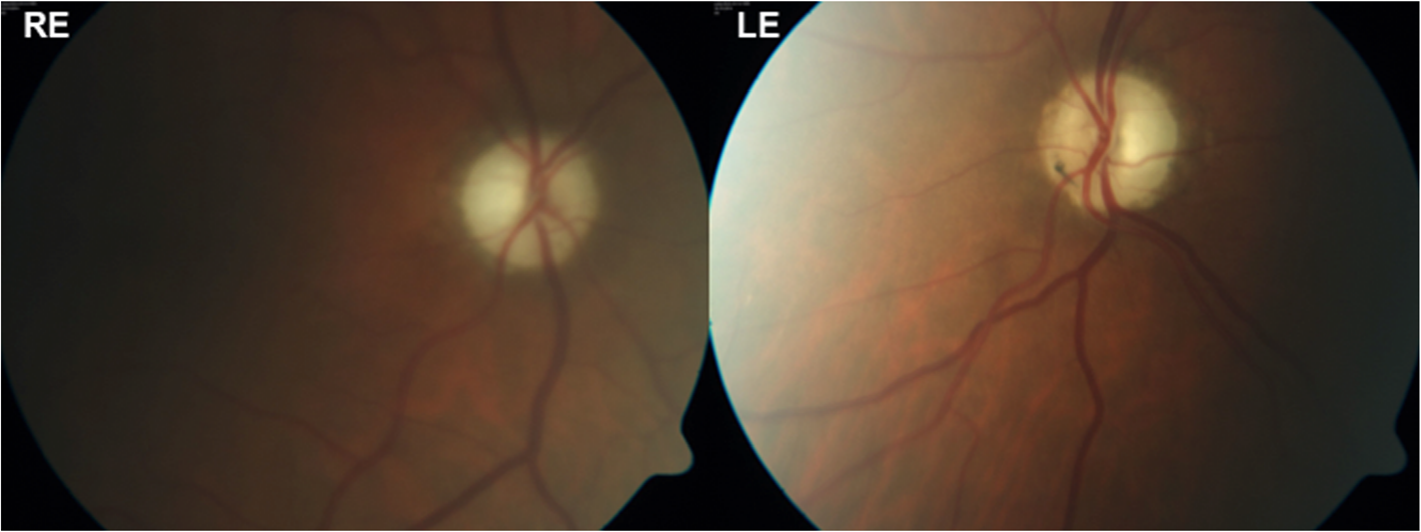
Patient 4, Posterior pole fundus imaging (30°) showing pale optic discs bilaterally

A 55 year-old male had bilateral visual loss, following surgery for aortic valve insufficiency. His previous ophthalmological check-up, revealed no pathology. During a mini-sternotomy the mammary artery has been injured, leading to hemodynamic shock. His visual acuity four days postoperatively was accessed as no light perception, with afferent pupillary defect bilaterally, and dilated pupils to 5.1 and 5.4 mm (Fig. 5). A few weeks later, the optic discs were found to be pale (Fig. 6), with severe reduction of the peripapillary retinal fiber layer thickness. Visual evoked potentials were not recordable. He was diagnosed with bilateral ischemic optic neuropathy, resulting from hypovolemic shock. A type 2 diabetes, hyperlipidemia and obesity were considered as systemic vascular risk factors. However, in addition, the patient reported a positive history of pre-existing six out of ten signs for FS (Table 2). The blood pressure drop was obviously a main factor for the bilateral optic nerve ischemia [7, 8], while the sympathomimetic drugs used to increase his blood pressure may have induced additionally a vasoconstriction of the ocular vessels, particularly in a patient with FS.

## Conclusions

We describe four patients with acute visual loss, noticed by the patients immediately after a surgery or a drug application. The literature reports visual loss after a variety of nonocular surgeries, among which spinal surgery, cardiac surgery and radical neck dissection held the greatest risk for this devastating complication [1, 2, 9, 10].

The final steps in the pathogenetic chain of events of perioperative visual loss in adults are mainly retinal vascular occlusion, ischemic optic neuropathy and ischemic cerebral lesions [3, 11]. High sensitivity of the retina to hypoxia, precipitated by hemodilution, may play a crucial role, particularly if the blood loss is significant and if the blood is replaced by crystalloid and colloid fluids only, rather than being complemented by blood transfusions or packed red blood cells.

The roles of intraoperative arterial hypotension/hypertension or increased IOP with consecutive reduction of the ocular perfusion pressure have also been discussed.

In our case series, low perfusion pressure, combined with disturbed autoregulation of the ocular blood flow, together with increased drug sensitivity seem to have played a central role in the pathogenesis of ocular hypoxia. The perfusion pressure (PP) of the eye depends on the difference between the mean arterial pressure and the retinal venous pressure (RVP). In healthy subjects, RVP is equal or slightly above the IOP. However, in patients with Flammer syndrome RVP is often distinctly above the IOP [12]. Therefore, the PP is lower than estimated just on Blood Pressure and IOP alone [13]. According to the anesthetic protocol, the MAP of Patient 1 was consistently around the lower limit of the normal range throughout the entire surgery. Together with an increased IOP (as for instance, when Ketamine is used) and an even higher RVP, PP can drop to very low values which are particularly relevant if at the same time the autoregulation is disturbed. A potential influence of a mechanical compression of the eye in prone position during scoliosis surgery (Patient 1) or during peribulbar steroid application (Patient 3) can not be ruled out. Postoperatively fundus examination revealed ocular hypoxia, which involved the retina (Patient 1), the optic nerve head (Patients 1, 3 and 4) and the choroid (Patients 1 and 4).

### The trilateral link between anaesthesia, perioperative visual loss and Flammer syndrome

All four patients reported visual loss immediately after systemic surgery, ophthalmic surgery, or ocular interventions, while all of them suffered simultaneously from Flammer syndrome. Our hypothesis is that FS may increase the risk for perioperative visual loss, and is based on the following facts:

1. As it is now well established, and further exemplified in our case series, that subjects with Flammer syndrome have intrinsically insufficient and unstable blood flow autoregulation.
2. Flammer syndrome subjects tend to react to a number of stimuli, such as cold, physical or emotional stress, and systemic medication, like adrenalin, with marked vasoconstriction [14, 15]. The most noteworthy problem in FS subjects seems to be an arterial vasospasm [16].
3. Subjects with FS often suffer from systemic hypotension [14], partly due to reduced sodium reabsorption in the proximal tubules of the kidneys [17, 18]. The latter in turn is due to prostaglandin E2 release, following increased systemic or local production of endothelin-1.
4. Subjects with FS often exhibit further blood pressure dips; therefore they have a lower perfusion pressure.
5. Subjects with FS have impaired autoregulation of their ocular blood flow, in which vascular endotheliopathy plays an important role [14].
6. Subjects with FS have, on average, higher retinal venous pressure [12, 19]. This contributes further to a decrease of the perfusion pressure in the central retinal vein and in the optic nerve head, which has its venous outflow again via the central retinal vein.
7. Subjects with FS have altered drug sensitivity, which is linked to altered expression of the ABC transport proteins [20]. This in turn may lead to yet unknown side effects during anaesthesia.

To note well, the complete vascular workup in our patients was positive for history of cold extremities, increased reactivity to cold (in all patients), and findings of increased plasma Endothelin-1 levels (Patient 1). Furthermore, the capillary blood flow cessation, registered through Nailfold Capillaroscopy with cold provocation test (Patients 1, 2 and 3) [22] and the other diagnostic signs (Table 2) [23] pointed toward unstable blood flow autoregulation as part of Flammer syndrome.

We will summarise here some prominent signs and symptoms, in order to complete the profile of such patients. Subjects with FS tend to have cold extremities [14] and reduced feeling of thirst [21]. They require longer time to fall asleep, especially when they are cold [22], as warm feet are generally a prerequisite for falling asleep. FS subjects also suffer more often from atypical headaches and migraines [23] and have increased pain sensations, since ET-1 decreases the pain perception threshold [24]. The flow cessation in nailfold capillaries after cold provocation is prolonged [23]. Reportedly, they have altered gene expression in the circulating lymphocytes [25] and slightly increased plasma Endothelin-1 level [21]. The syndrome occurs more often in females than in men, more often in thin than in obese, and - in young than in older subjects [26]. The systemic oxidative stress in such individuals is increased, most probably due to the unstable blood flow.

In all of the presented patients the vascular dysregulation dynamics were supposedly influenced by pharmaceuticals, with known side effects.

The pharmacological agents used during the surgery of Patient 1 were: Fentanyl, Remifentanil, Morphine, Thiopental, Atracurium, Ketamine, Propofol, Ringer’s lactate, HAES, Cefazolin, Paracetamol and Oxygen. Hypotension is observed after Morphine and Remifentanil medication. Thiopental and Atracurium may have hypotonic effect, mediated by histamine release [27, 28]. Ketamine has a well-pronounced hypertensive effect and is also known to increase the IOP, thus negatively influencing the ocular blood flow by decreasing the perfusion pressure.

Raynaud-like phenomenon has been described in a 14-year-old girl after Propofol medication [28]. Cerebral vasoconstriction has also been reported with Propofol intake [29].

In Patient 2 the anaesthetic protocol comprised Meperidine, Propofol, Ringer’s lactate, Hydroxyethyl Starch (HAES), Cefazolin, Paracetamol and Oxygen. Here the medications given to an individual with pre-existing Flammer syndrome and normal tension glaucoma produced the so dreaded outcome of visual loss. On the contrary, to our knowledge, a betamethasone-vascular dysregulation interaction (patient 3) has not been reported so far [29, 30].

Regarding Patient 4, during cardiac surgery a catecholamine (Norepinephrine) is routinely used to preserve the vessel tonus under open-air conditions. However, Norepinephrine is known to produce peripheral vasoconstriction [7].

Studying four patients with Flammer syndrome, in which seemingly different influences exacerbated the vascular disturbance, increased our understanding of this condition and the precautions to be taken, if a surgery or an invasive treatment, such as peribulbar or retrobulbar medication is to be performed.

Our findings shed new light on the pathogenesis of perioperative visual loss, while we ought to point out the limitations of our study, stemming from the small number of observed patients. While thankfully this complication is quite rare, further reports and observational prospective studies would be necessary, in order to establish the rate of perioperative visual loss among subject with FS, compared to the general population undergoing anaesthesia and surgery, and how the perioperative management of such subjects can promote a more favourable outcome in terms of vision preservation.

In Summary, we observed perioperative visual loss in four consecutive patients, and all data pointed toward episodes of ocular hypoxia. All four patients also had Flammer syndrome, therefore we presume that the syndrome may distinctly increase the risk for this devastating complication. A preoperative screening for FS would identify the patients in need of preventive measures. The screening can be done through thorough history taking, sensitive for data pointing toward probability for Flammer syndrome, such as prominent cold sensitivity, increased sleep latency, low blood pressure and other features of the syndrome (Table 2).

Identifying patients with FS may allow undertaking preventive measures, such as avoidance of: low blood pressure, cold provocation and vasoconstrictors, while implementing pre-treatment with magnesium [31] and low dose Calcium-channel blockers [14]. Calcium-channel blockers in adapted lower doses are preferred for their higher lipid affinity, which leads to better penetration through cerebral vessels, as well as for their milder effect on retinal vessels. Very productive and cost-effective approach to a patient with known or presumed FS is to administer omega-3 fatty acids supplementation and/or a diet containing them [32, 33], together with magnesium preparations, during the last two weeks preceding the surgical treatment.

Finally, in our case series, we assume a disturbed blood flow autoregulation, and a hyperreactivity toward pharmacological stimuli, to be the etiological bases of the unstable blood supply during the respective interventions. This process leads to significant and rapid increase of free radicals and enormous oxidative stress in individuals with Flammer syndrome. In our patients, based on all presented findings, we also hypothesise a hyperactivity of the sympathetic nervous system during or immediately after the surgery, or procedure, which resulted in massive vasospasm of the retinal, choroidal and/or peribulbar vessels, thus producing a visual loss.

The presented herein in-depth analysis of vascular dysregulation factors in a diverse group of patients, including an adolescent individual, has promoted our understanding of the pathological mechanisms leading to visual loss after uncomplicated (from systemic point of view) anesthesia or surgery, and was crucial for identifying effective preventive strategies.

## Ethics approval and consent to participate

The study and data accumulation were in conformity with our institutional requirements (named “Ethikskommisssion Nordwest- und Zentralschweiz – EKNZ”) and in accordance with the Declaration of Helsinki, and all governmental regulations. A detailed explanation was given to the patients before each procedure and test, and the procedure or test was performed only after they gave their consent.

## Consent for publication

Written consent was obtained from the patients (and the parents of Patient 1) for publication of these Case reports and any accompanying images. Copies of the written consents are available for review by the Editor of this journal. The presented herein material preserves the anonymity of the patients.

## Abbreviations

a.: artery
aa.: arteries
°C: degree Celsius
DWI: Diffusion Weighted Imaging
EDTA: Ethylene Diamine Tetra-Acetic acid
ET-1: Endothelin-1
FS: Flammer syndrome
HAES: Hydroxyethyl Starch
IOP: intraocular pressure
MAP: mean arterial pressure
OCT: Optical Coherent Tomography
ONH: Optic nerve head
mmHg: millimeter of mercury
PP: perfusion pressure
RI: Resistance Index
RVP: retinal venous pressure
v.: vein
